# Research progress on the N gene of Akabane virus

**DOI:** 10.3389/fvets.2025.1690905

**Published:** 2025-09-24

**Authors:** Ruining Wang, Fang Liang, Xiaolin Lan, Gan Li, Feng Li, Mengmeng Zhao

**Affiliations:** ^1^College of Veterinary Medicine, Henan University of Animal Husbandry and Economy, Zhengzhou, China; ^2^Guangdong Provincial Key Laboratory of Animal Molecular Design and Precise Breeding, School of Animal Science and Technology, Foshan University, Foshan, China; ^3^Center of Animal Epidemic Disease Prevention Control, Chongzuo, Guangxi, China

**Keywords:** Akabane virus, N gene, viral replication, diagnostic methods, AKAV diagnostics

## Abstract

Akabane disease, an arthropod-borne viral infection transmitted by Culicoides mosquitoes, causes severe reproductive disorders in livestock, including abortion, stillbirth, and congenital arthrogryposis-hydranencephaly syndrome. Caused by Akabane virus (AKAV), a single-stranded negative-sense RNA virus of the genus *Orthobunyavirus* (family *Peribunyaviridae*), this pathogen poses significant economic threats to global cattle and sheep industries. This review comprehensively examines the nucleocapsid protein (N) encoded by the AKAV S segment, which forms a conserved ribonucleoprotein (RNP) complex essential for viral genome protection, replication, and transcription. The structural characteristics of the N gene were analyzed, its minimal genetic variation across genotypes (97–100% homology), and functional roles in viral pathogenesis. Furthermore, applications of the N gene in diagnostic development (e.g., ELISA, PCR, colloidal gold immunoassays) and vaccine design were summarized, highlighting its utility as an immunogenic target due to high conservation and early antibody in-duction. By integrating these genetic, structural, and applied research advances, this review provides a theoretical foundation for novel control strategies against AKAV.

## 1 Introduction

Akabane disease is a polymorphic infectious disease caused by the Akabane virus (AKAV) (1). It affects cattle, sheep, and other domestic animals (2, 3), including camels and horses (4). In recent years, infections in bamboo rats have also been reported (5, 6). The virus was first prevalent in Chiyu Village, Gunma County, Japan in 1949, and it was not until 1959 that the pathogen was isolated from *Culex pipiens* aurea and Culex tritaeniorhynchus for the first time (7–9), and then cases were reported in many parts of the world. In China, AKAV antibodies were detected in cattle and sheep sera by the Animal Quarantine Institute of the Ministry of Agriculture in 1990 (10). In 1994, the presence of AKAV was first demonstrated in a seroepidemiologic survey conducted by Li et al. (11). In 1998, AKAV was isolated for the first time by Li et al. (12), and the physical and chemical characteristics and morphological and serological properties of the virus were initially characterized.

**Figure 1:**
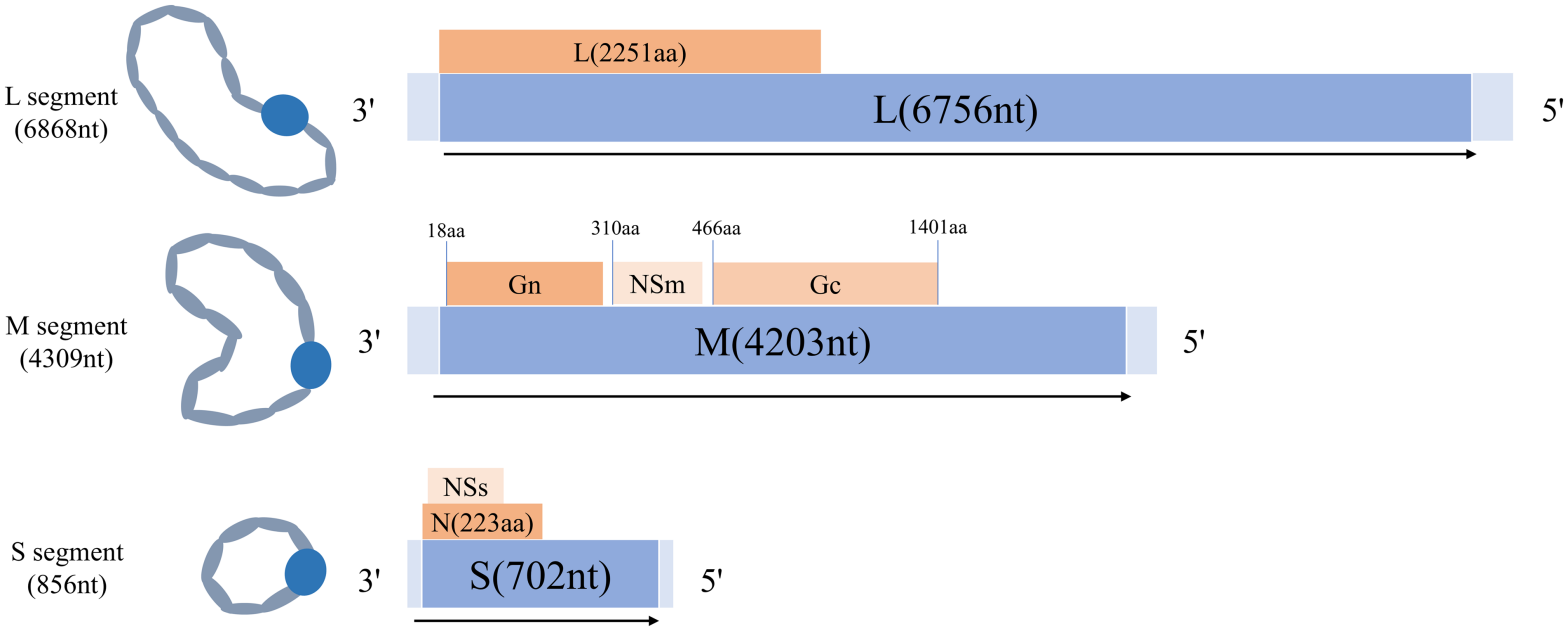
Schematic diagram of the gene structure of AKAV.

AKAV belongs to the family *Bunyaviridae*, the genus *Orthobunyavirus*, the Simbu serogroup, and is a single-stranded negative stranded RNA virus (13). The transmission of AKAV is largely dependent on arthropods such as mosquitoes and *Culex pipiens* (14–17). They transmit the virus by biting domestic animals (18, 19). Clinical signs of AKAV infection are diverse, varying but intersecting at different stages of gestation, and include abortion, preterm labor, stillbirth, fetal malformations, dyskinesia in calves, and congenital curvature of joints. Infection with AKAV can lead to a variety of symptoms, such as premature birth, stillbirth, fetal dyskinesia, congenital joint curvature [8], and non-purulent encephalitis (20), congenital brain abnormalities (21, 22), hydrocephalic anencephaly (Arthrogryposis Hydraencephaly, AH syndrome) (23, 24). The economic losses caused by AKAV are particularly severe in the cattle and sheep industries. The spread of AKAV is obviously seasonal, periodic and regional (25), so it is very important to monitor and take measures to prevent AKAV. The genome of AKAV consists of L, M, and S segments (26), the L segment encodes RNA polymerase (RdRp protein), the M segment encodes two vesicle membrane glycoproteins (Gc and Gn) as well as nonstructural proteins (NSm), and the S segment encodes nucleocapsid proteins (N) and nonstructural proteins (NSs). The structural pattern diagram was shown in Figure 1, which is helpful for understanding the structural composition of the AKAV. As the main nucleocapsid protein of AKAV, N protein is highly conserved (27) and immunogenic, and it is an important marker for early diagnosis after virus infection (28, 29).

In AKAV research, the characteristics of N gene have attracted wide attention. N genes are not only abundant in virus particles, but also highly expressed in infected cells (4). In addition, N genes have high similarity among different virus strains (30), which is an ideal antigen target for establishing type-specific detection methods. In recent years, monoclonal antibody (MAb) prepared from the N gene provides a new tool for the detection in AKAV (31). In this minireview, research progress of AKAV N gene, including its role in virus replication and transcription, and the development and application of diagnostic methods based on N gene are reviewed. Through the comprehensive angenesis of N genes, the purpose is to lay a foundation for the prevention, control and treatment of AKAV.

## 2 Structure and properties of the N gene

AKAV particles are approximately spherical and icosahedral, with diameters ranging from 70 to 110 nm, and possess a capsid and protein fibrils (32). Negative staining with phosphotungstic acid shows that the surface of the capsule has glycoprotein protrusions (33). AKAV is susceptible to lipid solvents such as chloroform and ether and can be inactivated by 20% ether within a short time. The virus is not heat-resistant, can be quickly inactivated at 56 °C, and is sensitive to acid, so it is difficult to maintain structural stability at pH 3. The relative molecular weight of the virus is 3–4 × 10^8^, the sedimentation coefficient is 350–475 S, and the floating density in CsCl is 1.29 g/cm^3^ (34). AKAV has erythrocyte agglutination (HA) and hemolysis (HL) under certain conditions and can agglutinate red blood cells of pigeons and geese under high salinity and pH 5.9. Pigeons can have hemolysis after agglutination (35), but do not agglutinate red blood cells of mammals such as human, sheep, cattle and guinea pigs (36).

In the chicken embryo model infected by AKAV, embryonic dysplasia and musculoskeletal malformation can be observed (37). Pure-bred mice are ideal experimental models. Neurological symptoms can be observed by inoculating the virus in the brain of mice, and then the virus passages can stably lead to the death of mice (4). AKAV shows strong affinity to fetal brain and skeletal muscle tissue (4). AKAV has a wide range of cytotropism, and the virus can proliferate in many kinds of cells, among which HmLu-1, Vero and BHK-2L are the most susceptible (38), and after inoculation, obvious cytopathy and plaque formation occur (4).

N gene is a structural protein encoded by S segment, and it is the nucleocapsid protein of virus (39), with tetrameric structure and molecular weight of 19–26 KD. N gene has three epitopes and group specificity, which can induce the body to produce antibodies (40). The main function of N gene is to wrap the virus gene and form irregular, circular and spiral ribonucleoprotein (RNP) (41), thus protecting the virus genome and preventing the genetic information from being destroyed by the host defence mechanisms.

## 3 N gene inheritance and variation analysis

AKAV are segmented RNA viruses, exhibiting significant variability and recombination (42). Evolutionary tree analysis of the S and M segments of the AKAV genome showed that AKAV is mainly divided into four genotypes (I–IV), of which the gene I-type is subdivided into Ia and Ib genotypes (43). The AKAV Ia genotype is mainly distributed in China, Korea, and Japan (43, 44). The AKAV Ib genotype is mainly distributed in Korea, Japan, Israel, and Turkey (45–47). AKAV genotype II is mainly distributed in Japan and Korea (48, 49). Genotype III is mainly found in Australia, and genotype (50). IV is mainly found in Africa (51). Genotype I is neurotropic and causes reproductive disorders and encephalitis (52). The lirki strain causes fatal non-purulent encephalomyelitis in newborn cattle (53). Genotype II is often caused by intrauterine infection and is characterized by abnormal parturition (27). The OBE-1 strain causes severe fetal malformations (53). The similarity between different genotypes of AKAV is very high, up to 93–100% (54), so there is a strong cross-reaction between them.

Among the three segments of AKAV, S segment and L segment are relatively conservative, while M segment is highly specific. Studies have shown that the similarity between N gene of 23 AKAV strains is 97–100% (30), and N gene are highly conservative. The results of ELISA constructed with N gene monoclonal antibody showed that it had high reactivity to all AKAV isolates (27). The conservation of N gene is not only reflected in different AKAV strains, but also in Schmallenberg virus (SBV), which is also a Simbu serogroup. The similarity between AKAV and SBV N genes is as high as 77% (54), and the antibodies produced by their N gene also have obvious cross-reaction. The detection method established for AKAV N gene antibodies can also detect SBV (54). Current studies have utilized the insect Drosophila S2 cell expression system to produce recombinant GcH protein and established an ELISA detection method that can differentiate between AKAV and SBV. This detection method exhibits significantly higher specificity than the ELISA method based on N gene and is faster, safer, and more suitable for high-throughput detection compared to the serum neutralization test (SNT) (55).

A total of 44 nucleotide sequences of the AKAV N gene were selected for phylogenetic analysis. The constructed phylogenetic tree revealed that Chinese strains formed relatively concentrated and distinct branches, demonstrating a certain degree of genetic independence. A closer phylogenetic relationship was observed between some Chinese strains and those isolated from Japan, suggesting possible historical gene flow or shared evolutionary origins. Furthermore, the formation of relatively independent clusters among Chinese strains indicated distinct evolutionary pathways, which may have been driven by local environmental adaptations and other factors during genetic evolution (Supplementary Figure S1).

## 4 Role of N genes in viral replication and transcription

The N gene collaborates to form the RNP complex by binding to viral negative strand RNA (vRNA). This RNP complex not only shields the AKAV genome from degradation by intracellular nucleases but also furnishes a structural framework for RNA replication and transcription (56). This is essential for RNA replication and transcription. Wang et al. (38) found that AKAV replication is inhibited at the indicated time points in cell lines constitutively expressing AKAV N gene by studying two positive BHK-21 cell lines stably expressing EGFP (named C8H2 and F7E5). Subsequent analysis by growth kinetics and qRT-PCR showed that stable overexpression of AKAV N gene in BHK-21 cells could temporarily inhibit viral replication by reducing viral mRNA expression. It was also found that stable overexpression of AKAV N gene in constructed cell lines could restore replication-deficient AKAV strains, which could be achieved by co-transfecting plasmids carrying AKAV-L, AKAV-M and AKAV-S4N fragments into C8H2 or F7E5 cells. The AKAV virosome can then be assembled with the help of the cell-expressed AKAV N gene. The rescued AKAV strains can only propagate in C8H2 or F7E5 cells and are replication-deficient in other cells, a feature that could provide new samples for the development of AKAV vaccines.

**Figure 2:**
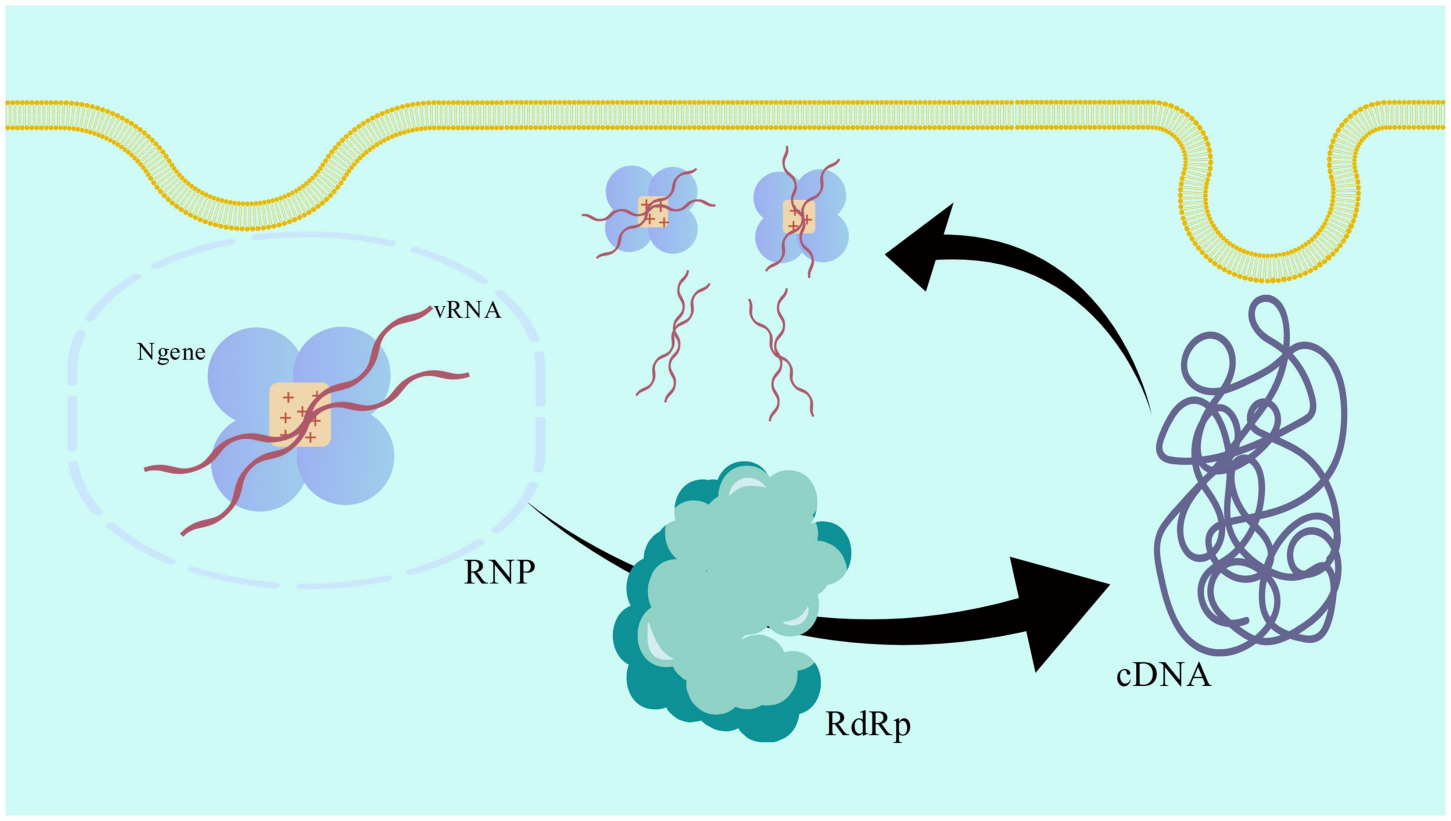
Schematic diagram of the transcription and replication cycle of the viral RNP complex. The pattern was created with BioGDP.com.

In the first step of viral transcription, the N gene forms a continuous positive charge groove at the inner ring of its tetramer structure and forms an RNP complex by closely combining with vRNA in a cross configuration (57). As a template of viral RNA polymerase, the RNP complex promotes the synthesis of complementary plus-strand RNA (cRNA). In the primary transcription stage, RdRp uses vRNA as a template to synthesize cRNA. N genes can increase the efficiency and accuracy of RdRp by stabilizing the vRNA template, thus ensuring the effective synthesis of cRNA. Through the replication activity of RdRp, the newly synthesized cRNA can be used as a template to synthesize more vRNA. In this process, N gene continues to protect the cRNA template and prevent it from being degraded by nuclease in the host cell. In the process of replication, the newly synthesized vRNA combines with N gene again to form a new RNP complex. These RNP complexes can be used for further transcription and replication, or as part of the assembly of new virus particles (58). The schematic diagram of the process is shown in Figure 2, which clearly demonstrates this cyclic process (59).

In the study by Norasuthi Bangphoomi (60) on AKAV’s entry into mammalian cell lines, as well as in the research conducted by Shin Murakami (53) on the replication and adsorption of AKAV and SBV in susceptible cells with heparan sulfate proteoglycans (HSPGs), the N gene has served as an important marker for assessing the interactions of viral entry into cells and the mechanisms of replication and transcription.

## 5 Application of N gene in diagnosis

The currently known commercialized vaccine for AKAV is the trivalent inactivated vaccine for abnormal deliveries in cattle of Akabane virus, Aino virus (AINOV), and Chuzan virus (CHUV), manufactured by Nissin Co (55, 61) and DS Akabane Cattle Vac manufactured by Daesung Microbiological Labs, Gyeongido Korea, Ltd. (62). The N gene of AKAV is recognized as one of the most highly conserved structural proteins within the Simbu serogroup (38), which provides a foundation for the development of N-based vaccines with the potential to confer cross-strain protection. The ability of mAb 1H4 to bind to nearly all tested strains of Simbu viruses further supports the rationale for targeting the N protein as a broad-spectrum vaccine candidate (63). Additionally, the N gene can be efficiently expressed in bacterial expression systems, making it suitable for large-scale vaccine production and quality control (64). An early and robust antibody response is induced by the N protein during infection, which may contribute to the rapid activation of the host immune system (65). However, it should be noted that the specific antibodies generated in response to the N protein lack virus-neutralizing activity (63). This indicates that while N protein-induced antibodies are valuable for diagnostic purposes, they are unable to directly neutralize the virus. Consequently, vaccines based on the N protein may not provide effective infection-blocking protection and might be more appropriate as post-infection viral clearance agents or immune enhancers. Furthermore, the use of such vaccines could complicate the differentiation between vaccinated and infected animals.

At present, there are many methods to detect AKAV, Yang Su et al. (66) selected the nucleic acid sequence of AKAV N gene fragments by molecular cloning method, and used a microarray sampler to spot sample onto coated glass slides, and finally produced a microarray capable of simultaneously detecting the pathogens of five infectious diseases, including foot-and-mouth disease virus, vesicular stomatitis virus, bluetongue virus, deer epidemiological hemorrhagic fever virus, and AKAV. Liu et al. prepared AKAV N protein expression using a prokaryotic expression system, laying the foundation for the establishment of subsequent detection methods. Li and others (4, 39, 54, 67, 68) establish AKAV ELISA detection methods, respectively. Among them, Li established the first ELISA method, which was found to be 2 times more sensitive than the serum neutralization (SN) test and 16 times more sensitive than the agar gel precipitation (AGP) test. Furthermore, Li preliminarily developed an indirect ELISA kit, and it was demonstrated that the sensitivity of the reconstituted kit remained unaffected after being subjected to accelerated degradation at 37 °C for 3 days; the indirect ELISA detection kit developed by Xu demonstrates that the recombinant antigen can be stored at −80 °C for 10 months without loss of activity, and low antibody titer samples can still be effectively detected; Wang encapsulated a recombinant N protein, which avoided the biological hazards of the whole virus; and Chen established an assay that could simultaneously detect AKAV and SBV. The assay was able to detect SBV at the same time, with the disadvantage of not being able to distinguish between AKAV and SBV. Chen (69) developed a double antibody sandwich ELISA (DAS-ELISA), which can detect a lower concentration of antigen, and the two antibodies are directed against different epitopes of the antigen, which reduces cross-reactivity and non-specific binding. Kong et al. (70) used anti-AKAV N protein rabbit polyclonal antibody to develop a colloidal gold immunoassay test strip for bovine Akabane disease. This detection method is not affected by the surrounding environment, does not require precision instruments for detection, is cost-effective, and is economical and environmentally friendly, making it suitable for large-scale sample detection in the field (71). Fang et al. (72) prepared monoclonal antibody against AKAN N protein, laying a foundation for further research on N protein and the detection of AKAV antigens.

As early as 2003, Hua et al. (73) constructed and expressed a fusion expression vector of AKAV N gene and thioredoxin, and the recombinant nuclear protein antigen produced by L-arabinose induction could replace the complete virus as a target for serological detection. Akashi et al. (74) constructed a nested PCR that can detect and distinguish between AKAV and AINO at the same time. The detection concentration is lower, and the operation is simpler. Takenaka-Uema et al. (75) used reverse genetics technology to artificially construct a bifunctional S genome, which codes for N/NSs protein on the negative strand and eGFP on the positive strand, separated by the Intergenic Region (IGR) derived from the Rift Valley fever virus (RVFV) S genome. Through virus rescue, they successfully rescued the recombinant virus eGFP-AKAV. This not only provides new research tools but also provides a new perspective for the pathological study of AKAV and lays a foundation for the diagnosis and prevention of AKAV.

## 6 Conclusion

Genetic variation analysis of the AKAV N gene has shed light on the evolutionary dynamics and geographic distribution patterns of AKAV. The identification of different genotypes and subtypes contributes to the understanding of virus transmission pathways and host adaptation. This information is a practical reference for preventing the spread of AKAV and developing epidemic prevention programs. The N gene also plays an indispensable role in viral replication and transcription (27, 55), not only it can bind to RNA to form RNP complexes to protect the integrity of the viral genome, but also the level of N gene expression affects the transcription and replication efficiency of the virus.

The AKAV N gene is widely used in detection and vaccine development due to its conservatism. The detection methods developed using the N gene have shown good specificity and sensitivity in both laboratories and clinical settings. The popularization and application of these methods have improved the detection efficiency and accuracy of AKAV, effectively curbing its spread. They also play an important role in under-standing the distribution and epidemiology of AKAV in China and contribute significantly to the healthy development of the cattle and sheep industries in China. At the same time, they lay a solid foundation for further research on AKAV. To date, AKAV continues to be prevalent globally, posing a serious threat to the development of the livestock industry. Research on the pathogenesis and genetic characteristics of AKAV can provide scientific basis for the prevention and control of the virus.
