# Supplementary material for: Research progress on the N gene of Akabane virus
Source: Front Vet Sci. 2025 Sep 24;12:1690905. doi: 10.3389/fvets.2025.1690905 (PMC12504090; doi:10.3389/fvets.2025.1690905)
Supplement: Supplementary file 1 [file Data_Sheet_1.PDF]

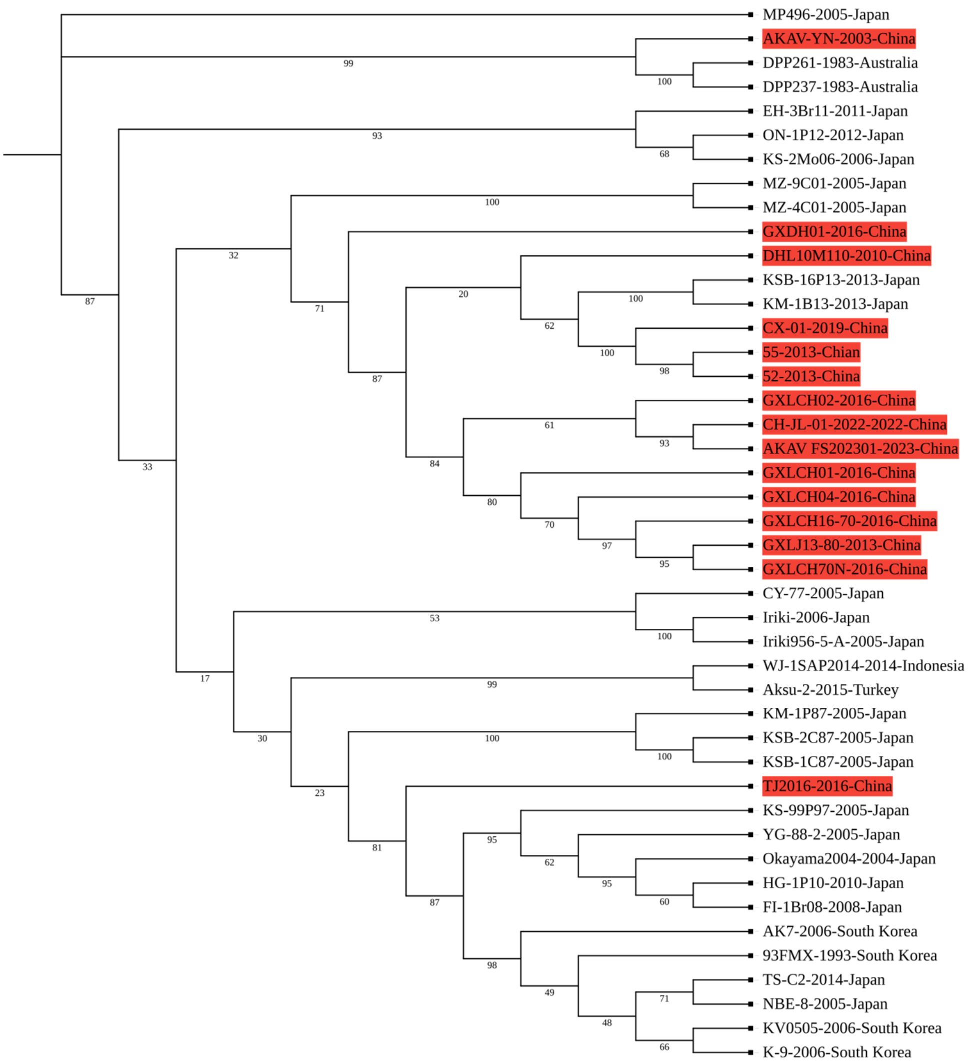

Figure S1. Evolutionary tree analysis of the AKAV N gene. 1000 bootstrap replicates were performed using the neighbor - joining (NJ) method in MEGA software (version 12.0).The Chinese AKAV N gene strain should be highlighted with a red background.
